# Effectiveness of Prothrombin Complex Concentrate Versus Fresh Frozen Plasma in Major Cardiac Surgeries: A Systematic Review and Meta-Analysis of Randomized Controlled Trials

**DOI:** 10.7759/cureus.94405

**Published:** 2025-10-12

**Authors:** Abdullah M Alharran, Taif M Alotaibi, Abdulaziz M Alazmi, Layal T Alwazzan, Maryam E Alawi, Maisam Elsadig, Saud A Alyahya, Abdallah M Shanat, Mubarak F Alajmi

**Affiliations:** 1 College of Medicine and Medical Sciences, Arabian Gulf University, Manama, BHR; 2 Faculty of Medicine, Ministry of Health, Kuwait City, KWT; 3 Faculty of Medicine, Royal College of Surgeons in Ireland, Busaiteen, BHR; 4 Faculty of Medicine, Ain Shams University, Cairo, EGY; 5 Faculty of Medicine, Royal College of Surgeons in Ireland, Dublin, IRL; 6 Faculty of Medicine, Jordan University of Science and Technology, Irbid, JOR

**Keywords:** cardiac surgery, ffp, fresh frozen plasma, pcc, prothrombin complex concentrate

## Abstract

Major bleeding is a common and important complication following major cardiac surgeries. Fresh frozen plasma (FFP) is the standard therapy for bleeding-related complications. Prothrombin complex concentrate (PCC) may offer a good alternative during cardiac surgeries. We aimed to assess whether PCC could potentially replace FFP in clinical practice. We searched different databases from inception until July 2025 for only randomized controlled trials (RCTs) that compared PCC with FFP in patients undergoing major cardiac surgeries and assessed our outcomes of interest in an intention-to-treat analysis. The primary outcome of interest was the change in international normalized ratio (INR). Data were pooled as risk ratio (RR) and mean difference (MD) with their 95% confidence intervals (CIs). A total of six RCTs with 761 patients were included in the final analysis. Patients allocated to PCC had lower INR values compared to those allocated to FFP (MD = -0.13, 95% CI: -0.18 to -0.07, p < 0.001). Additionally, PCC administration was associated with less blood drained from chest tubes (MD = -157.06 mL, 95% CI: -252.92 to -61.19, p < 0.001), fewer transfused RBC units (MD = -0.95, 95% CI: -1.25 to -0.65, p < 0.001), and higher hemostatic effectiveness rates (RR = 1.18, 95% CI: 1.02 to 1.36, p = 0.03). No significant differences were observed regarding safety measures. In patients undergoing major cardiac surgeries, the use of PCC showed superior effectiveness over FFP regarding hemostatic efficacy and other related outcomes. Also, PCC demonstrated similar safety measures compared to FFP.

## Introduction and background

In patients undergoing major cardiac surgeries, the incidence rate of major bleeding necessitating the transfusion of blood products is around 15% [1], with a significant increase in morbidity and mortality [2]. Despite the multifactorial nature of excessive bleeding, one of the most contributing factors to its incidence during cardiac surgeries is the depletion of coagulation factors to the extent of impairing thrombin generation [3], thereby decreasing the rate of fibrin clot formation [4].

Fresh frozen plasma (FFP) is the mainstay of therapy for bleeding complications as it contains the full components of procoagulant and anticoagulant factors [5] and is administered to around 30% of patients undergoing major cardiac surgeries [6]. Despite the widespread use of FFP in cardiac surgeries, there is a lack of robust evidence on its hemostatic efficacy in controlling coagulopathic bleeding, which can sometimes lead to life-threatening complications such as transfusion-related acute lung injury or serious allergic reactions [5,7].

Moreover, prothrombin complex concentrate (PCC) is derived from plasma and contains the main vitamin K-dependent coagulation factors, in addition to anticoagulant proteins C and S and a small amount of heparin, all of which can be used as an alternative to FFP [8]. The superiority of PCC over FFP lies in the fact that PCC undergoes purification, concentration, and pathogen reduction, and it does not require thawing or ABO matching [9]. However, since PCC does not include all coagulation factors found in FFP, there is uncertainty regarding its effectiveness in restoring hemostasis in cardiac surgical patients.

We aimed to study the relative effectiveness of PCC compared to FFP in terms of hemostatic factors and safety measures in patients undergoing cardiac surgery.

## Review

Methods

We adhered to the guidelines proposed by the Preferred Reporting Items for Systematic Reviews and Meta-Analyses (PRISMA) [10] and the statements of the Cochrane Handbook for Systematic Reviews of Interventions [11] during all stages of this systematic review and meta-analysis. This systematic review and meta-analysis was registered in PROSPERO (CRD420251140226).

Literature Search, Screening, and Eligibility Criteria

We conducted an electronic search on PubMed, Medline, Scopus, Web of Science (WOS), and the Cochrane CENTRAL from inception until July 2025 using the following search strategy: (((Prothrombin Complex Concentrate) OR (Coagulation Factor IX) OR (Plasma Thromboplastin Component) OR (Factor IX Complex) OR (Autoprothrombin II)) AND ((Fresh Frozen Plasma) OR (Blood Plasma)) AND (Cardiac Surgeries))). Detailed search terms for each database are shown in the Appendices. We further performed manual backward and forward citation analyses of the references of all included studies for any potentially relevant studies. We considered only studies published in the English language, without applying any additional filters.

All included citations were screened in two steps. First, we filtered the citations based on titles and abstracts, and then a full-text screening of all eligible studies was conducted according to our inclusion criteria. We used Rayyan (Rayyan Systems Inc., Cambridge, Massachusetts) for the screening process [12].

We considered only randomized controlled trials (RCTs) that included patients undergoing major cardiac surgeries with cardiopulmonary bypass (CPB) allocated to PCC as the intervention of interest and FFP as the main comparator. The studies had to assess our outcomes of interest, which were the change in international normalized ratio (INR) from baseline to 24 hours postoperation in an intention-to-treat analysis. Our main exclusion criteria were studies involving patients with a history of heart transplantation or a ventricular assist device. We also excluded studies with no relevant or unpublished data. Additionally, conference abstracts, observational data, and non-peer-reviewed data were excluded.

Outcomes

The primary outcome of interest was the international normalized ratio (INR). Additionally, the secondary outcomes of interest were chest tube drainage, volume of red blood cell (RBC) transfusion and number of patients requiring RBC transfusion, volume of allogeneic transfusions, hemostatic effectiveness, hospital and intensive care unit (ICU) length of stay (LOS), and safety outcomes, including rates of 30-day mortality, reoperation for bleeding, thromboembolic events, stroke or transient ischemic attack (TIA), and acute kidney injury (AKI).

Quality Assessment

Two authors independently assessed the risk of bias using the Cochrane Risk of Bias 2 (ROB-2) tool for RCTs [13]. The tool adopts five domains accounting for potential methodological differences in bias, with the main domains being selection, performance, detection, attrition, and reporting biases. The decision for each domain was labeled as high risk, some concerns, or low risk, and an overall risk of bias was assigned based on each domain's judgment. Any disagreements between authors were resolved through discussion with another author.

Data Extraction and Meta-Analysis

A standardized Excel data sheet was used to extract relevant data from the included studies. The selected data covered four domains: baseline characteristics of the cardiac surgical patients, including previous medical history, preoperative laboratory measurements, type of procedure, and procedural characteristics; summary of the included studies, including follow-up duration, inclusion and exclusion criteria, and overall findings; risk of bias domains; and outcomes of interest, including primary and secondary outcomes.

Dichotomous data were presented as the number of events and total patients and were further pooled as risk ratio (RR) with 95% confidence intervals (CIs) in an intention-to-treat (ITT) analysis. Data of patients lost to follow-up were imputed using the best-case scenario of the ITT. The DerSimonian-Laird random-effects model was used for the analysis. In cases of multiple follow-up points for the same outcome, we used data from the last follow-up point. Continuous data were presented as mean change, subtracting postmean values from premean values within the same group, and were pooled as mean difference (MD) with 95% CI using the DerSimonian-Laird random-effects model as the model of interest.

Heterogeneity was assessed using the Q test proposed by Cochrane and the I² measure across all included studies. Significant heterogeneity was defined as an I² value ≥50% and a p value <0.05. In cases of significant heterogeneity, a Galbraith plot was used to visualize studies with heterogeneous data relative to others [14]. A leave-one-out sensitivity analysis was also performed to test for small-study effects by excluding each study one at a time [15]. Additionally, a Doi plot was performed by measuring the LFK (Luis Furuya-Kanamori) index to test for publication bias [16]. STATA 19MP was used to conduct all statistical analyses using the “meta esize” and “meta forest” packages.

Results

Search Results

**Figure 1:**
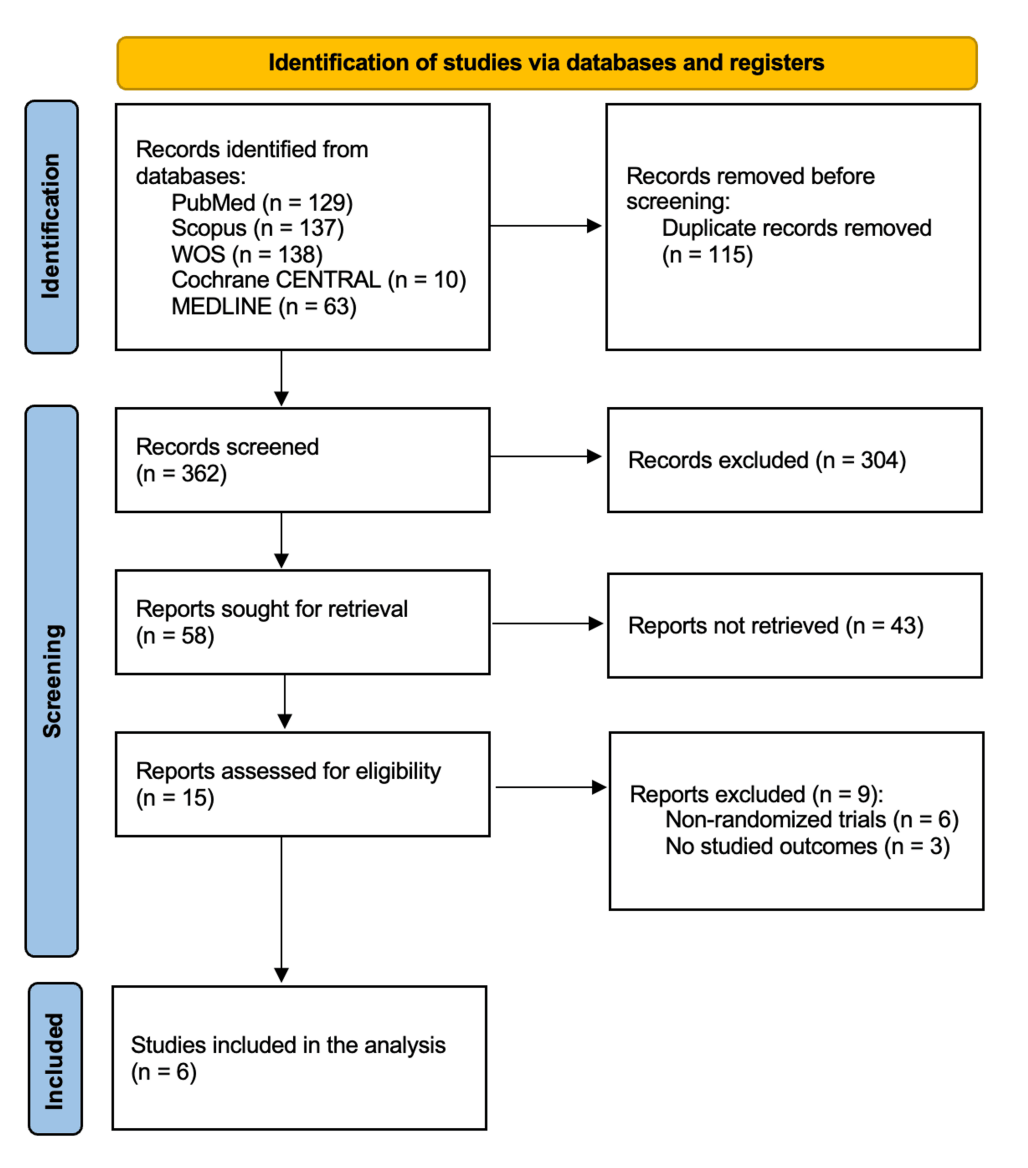
The PRISMA flow diagram of the selection process. PRISMA: Preferred Reporting Items for Systematic Reviews and Meta-Analyses.

The search across different databases yielded 477 citations, of which 115 were removed after duplicate removal and screening of titles and abstracts. Finally, a total of six RCTs [9,17-21] were included in the final analysis following full-text screening. The PRISMA flowchart is shown in Figure 1. 

Characteristics and Quality Assessment of the Included Studies

A total of six RCTs with 761 patients were included in the final analysis. The mean age of the patients was 67.6 years in the PCC group, while it was 66.7 years in the FFP group. Male patients numbered 523 (68.7%): 259 (49.5%) in the PCC group and 264 (50.5%) in the FFP group. All medical histories, comorbidities, and preoperative laboratory results were similar between patients in both groups. Detailed baseline characteristics of the included patients and summaries of the included studies are presented in Tables 1, 2. All included RCTs were assessed using the ROB-2 tool. Three studies had an overall low risk of bias, while the remaining three had some concerns, as shown in the Appendices.

**Table 1:** Baseline characteristics of the included patients. Data presented as n (%) or mean (SD). PCC, prothrombin complex concentrates; FFP, fresh frozen plasma; DM, diabetes mellitus; HTN, hypertension; TIA, transient ischemic attack; PVD, peripheral vascular disease; CHF, congestive heart failure; INR, international normalized ratio; CPB, cardiopulmonary bypass duration; CABG, coronary artery bypass grafting; NR, not reported.

Study	﻿Demeyere et al. 2010 [17]		﻿Farsad et al. 2015 [18]		Green et al. 2021 [19]		﻿Karkouti et al. 2021 [9]		﻿Smith et al. 2022 [20]		﻿Karkouti et al. 2025 [21]	
Group	PCC	FFP	PCC	FFP	PCC	FFP	PCC	FFP	PCC	FFP	PCC	FFP
Sample size	20	20	25	25	25	25	54	47	51	49	213	207
Age, years	69.6 (9)	71.2 (7)	66.7 (7.8)	65.8 (6.8)	69 (7.8)	66 (12.5)	66 (17.5)	67 (14.5)	70 (12.2)	69 (12.2)	67 (11.2)	64 (12.7)
Males	10 (50)	10 (50)	10 (40)	18 (72)	16 (64)	16 (64)	40 (74.1)	33 (70.2)	26 (51)	35 (71.4)	157 (73.7)	152 (73.4)
BMI, kg/m^2^	25.8 (3.9)	25.5 (3.9)	25.3 (3.9)	25.8 (4.3)	27 (6)	29 (5)	23.6 (4.5)	23.1 (4.7)	28.7 (6.2)	28.9 (5.2)	29 (6)	28 (5.4)
DM	NR	NR	NR	NR	6 (24)	6 (24)	11 (20.4)	10 (21.3)	NR	NR	48 (22.5)	45 (21.7)
HTN	NR	NR	25 (100)	25 (100)	19 (76)	13 (52)	36 (66.7)	31 (66)	NR	NR	144 (67.6)	141 (68.1)
﻿Atrial fibrillation	20 (100)	20 (100)	NR	NR	NR	NR	8 (14.8)	7 (14.9)	6 (11.8)	0	42 (19.7)	52 (25.1)
﻿Stroke/TIA	NR	NR	NR	NR	NR	NR	5 (9.3)	7 (14.9)	1 (2)	1 (2)	14 (6.6)	15 (7.2)
PVD	NR	NR	NR	NR	NR	NR	2 (3.7)	3 (6.4)	NR	NR	8 (3.8)	11 (5.3)
CHF	NR	NR	NR	NR	NR	NR	12 (22.2)	13 (27.7)	NR	NR	36 (16.9)	37 (17.9)
Previous cardiac surgery	NR	NR	25 (100)	25 (100)	2 (8)	1 (4)	19 (35.2)	11 (23.4)	NR	NR	53 (24.9)	56 (27.1)
Nonelective surgery	NR	NR	NR	NR	6 (24)	5 (20)	11 (20.4)	15 (31.9)	NR	NR	36 (16.9)	44 (21.3)
Complex surgery	NR	NR	NR	NR	NR	NR	45 (83.3)	37 (78.7)	NR	NR	144 (67.6)	152 (73.4)
Creatinine, mg/dL	NR	NR	1.08 (0.29)	1.14 (0.32)	NR	NR	1.1 (0.46)	1 (0.23)	NR	NR	0.96 (0.25)	0.95 (0.28)
Hemoglobin, g/dL	NR	NR	9.13 (1.2)	9.48 (1.19)	13.3 (1.3)	13 (1.8)	13.2 (1.9)	12.8 (2.1)	11.6 (1.5)	12.4 (1.5)	13.7 (1.9)	13.6 (2.02)
Platelet count, x 10^3^/μL	NR	NR	NR	NR	227 (51.1)	239 (94.3)	205 (58)	210 (67)	199.5 (64.1)	205.1 (70.6)	201 (53)	199 (60.4)
INR	2.8 (0.8)	2.8 (0.9)	4.02 (1.07)	4.8 (1.3)	NR	NR	1.2 (0.3)	1.2 (0.3)	1.75 (0.19)	1.73 (0.18)	1.1 (0.15)	1.1 (0.07)
CPB duration, min	114 (37)	121 (30)	NR	NR	NR	NR	172 (71)	166 (46)	161 (75.5)	155 (63.4)	171 (76.4)	176 (80.5)
Aortic valve	NR	NR	7 (28)	7 (28)	2 (8)	0	34 (63)	27 (57.4)	8 (15.7)	6 (12.2)	110 (51.6)	98 (47.3)
Surgery on the aorta	NR	NR	NR	NR	NR	NR	18 (34)	24 (51.1)	NR	NR	26 (12.2)	24 (11.6)
Mitral valve	NR	NR	10 (40)	7 (28)	NR	NR	17 (31.5)	16 (34)	NR	NR	48 (22.5)	47 (22.7)
Tricuspid valve	NR	NR	3 (12)	7 (28)	NR	NR	3 (5.6)	3 (6.4)	NR	NR	18 (8.5)	15 (7.2)
Pulmonary valve	NR	NR	0	0	NR	NR	7 (13)	1 (2.1)	NR	NR	6 (2.8)	7 (3.4)
Single valve	5 (25)	5 (25)	9 (36)	6 (24)	5 (20)	7 (28)	NR	NR	8 (15.7)	6 (12.2)	NR	NR
CABG	5 (25)	7 (35)	NR	NR	6 (24)	5 (20)	20 (37)	15 (31.9)	1 (2)	0	91 (42.7)	86 (41.5)
Congenital	NR	NR	NR	NR	NR	NR	6 (11.1)	3 (6.4)	0	1 (2)	NR	NR
Other	NR	NR	NR	NR	12 (48)	13 (52)	21 (38.9)	13 (27.7)	NR	NR	69 (32.4)	89 (43)
﻿Tranexamic acid dose, g	NR	NR	NR	NR	NR	NR	4.3 (2.3)	4.1 (1.3)	NR	NR	3.4 (1.6)	3.6 (4)
﻿Aminocaproic acid dose, mg	NR	NR	NR	NR	NR	NR	NR	NR	NR	NR	12.1 (5)	13.1 (5.5)
﻿Heparin dose, IU/mL	21,000 (NR)	21,000 (NR)	12,500 (NR)	12,500 (NR)	NR	NR	53,167 (18,107)	55,543 (20,996)	28,000 (NR)	27,040 (NR)	50,343 (20,288)	51,114 (21,474)
﻿Protamine dose, mg	NR	NR	NR	NR	NR	NR	405 (98)	451 (151)	270.4 (NR)	270.4 (NR)	381 (116)	390 (152)

**Table 2:** Summary of the included studies. PCC: prothrombin complex concentrate, FFP: fresh frozen plasma, INR: international normalized ratio, CPB: cardiopulmonary bypass, ECMO: extracorporeal membrane oxygenation, FP: frozen plasma, RCT: randomized controlled trial.

Author	﻿Demeyere et al. [17]	Farsad et al. [18]	Green et al. [19]	Karkouti et al. [9]	Smith et al. [20]	Karkouti et al. [21]
Year	2010	2015	2021	2021	2022	2025
Study type	RCT	RCT	RCT	RCT	RCT	RCT
Country	﻿Belgium	Iran	United Kingdom	Canada	USA	Canada and USA
Total number of patients	40	50	50	101	100	420
PCC, n	20	25	25	54	51	213
FFP, n	20	25	25	47	49	207
Inclusion criteria	Patients were enrolled after signing an informed consent if they met the following criteria: scheduled for urgent or semi-urgent cardiac surgery; under oral anticoagulation therapy with acenocoumarol, phenprocoumon or warfarin; INR ≥ 2.1, but without over-anticoagulation (INR > 7.8); and body weight ≤ 100 kg.	Patients with a weight under 100 kg, age between 18-85 years old, Warfarin consumption prior to intervention, initial international normalized ratio (INR) > 2.5, and consumption of FFP or PCC.	Adult patients (≥ 18 years) who were able to give consent and were undergoing elective or non-elective cardiac surgery.	Adult patients undergoing cardiac surgery for whom coagulation factor replacement with FP or PCC was ordered during surgery for management of bleeding were eligible for inclusion.	Adult patients aged 18 years and older undergoing cardiac surgery with the use of CPB were eligible to be screened for inclusion in the study. Patients undergoing complex cardiac surgical procedures (eg, aortic replacement surgery, multiple procedures, or repeated sternotomy) were preferentially targeted for enrollment. Patients provided written informed consent and were enrolled at the time of a preoperative visit.	Adult (≥18 years) patients undergoing cardiac surgery with cardiopulmonary bypass were eligible for inclusion in the study if the treating clinicians ordered coagulation factor replacement with PCC or frozen plasma for active or anticipated bleeding after termination of the cardiopulmonary bypass.
Exclusion criteria	Patients were not included in case of: renal or hepatic insufficiency, allergic reaction to blood products, disseminated intravascular coagulation, active thrombosis or pulmonary embolism, intracardiac thrombus, treatment with platelet inhibitors (except for aspirin), pregnant or breastfeeding women.	Patients with renal or hepatic insufficiency, allergic reaction to blood products, past history of Heparin-induced thrombocytopenia and disseminated intravascular coagulation, active thrombosis or pulmonary embolism, intracardiac thrombus, and pregnancy or breastfeeding.	Patients with bleeding risks, anticoagulant use, allergies, or recent thromboembolism. Also excluded: those undergoing specific surgeries, on ECMO, or recently in other clinical trials.	Patients receipt of FP or PCC within 48 hours before surgery; history of severe allergic reaction to FP or PCC; refusal of blood components; known pregnancy; anticipated high risk of death within 24 hours of surgery; undergoing heart transplantation, ventricular assist device implant or removal, or thoracoabdominal aneurysm repair; history of heparin-induced thrombocytopenia; receipt of warfarin with an international normalized ratio higher than 1.5 at the time of surgery; or receipt of direct oral anticoagulants within 48 hours of surgery.	Patients with fibrinogen level less than 144 mg/dL on initial post-surgery laboratory testing (to convert milligrams per deciliter to grams per liter, multiply by 0.01), life-threatening bleeding necessitating transfusion of hemostatic products (PCC or plasma) before the study intervention time point, circumstances in which the safety of the patient could be jeopardized by continued adherence to the study protocol, and extracorporeal membrane oxygenation requirement intraoperatively or postoperatively.	Patients with heart transplant, insertion or removal of ventricular assist devices, repair of a thoracoabdominal aneurysm, or any concomitant noncardiac surgery.
Results conclusion	PCC reverses anticoagulation safely, faster, and with less bleeding than FFP.	PCC is an effective and safe alternative to FFP in patients with mechanical heart valves. We would, therefore, recommend that PCC be employed for rapid and effective correction of Warfarin in patients with mechanical heart valves who require urgent Warfarin reversal.	PCC rapidly restored vitamin K-dependent factors with no increase in thromboembolic events compared to FFP.	Patients who require coagulation factor replacement for bleeding during cardiac surgery; this pilot study illustrates that a multicenter randomized trial comparing PCC with FP is feasible. Our results suggest that PCC may be a suitable substitute for FP because it markedly decreases the need for FP and may have hemostatic superiority without increasing the occurrence of adverse events.	The use of PCCs in patients undergoing cardiac surgery has become increasingly common. The off-label use of PCCs in the setting of coagulation factor–mediated post-CPB coagulopathy, however, has been cautiously accepted, owing to a somewhat unknown safety profile and a lack of prospective randomized clinical trials.	PCC had superior hemostatic efficacy and may have safety advantages over frozen plasma in patients who require coagulation factor replacement for bleeding during cardiac surgery. Given the magnitude of the treatment effect, preferentially using PCC over frozen plasma for bleeding management in cardiac surgery could have benefits for patients (by reducing bleeding and exposure to allogeneic blood products) and for the health care system (by relieving pressures on the blood supply and hospital resources).

Outcomes

Primary Outcome

**Figure 2:**
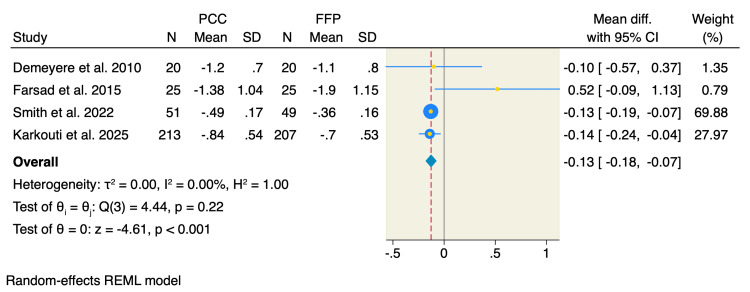
Random-effects model of mean change of international normalized ratio (INR). Data from [17,18,20,21].

**Figure 3:**
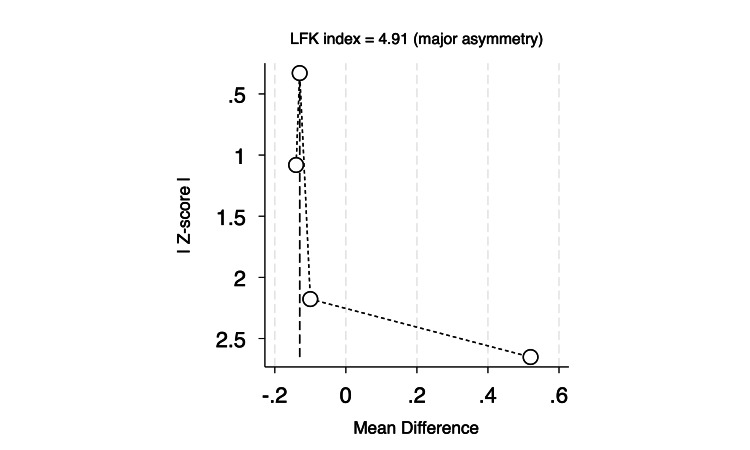
Doi plot (LFK index) of mean change of international normalized ratio (INR). LFK: Luis Furuya-Kanamori.

**Figure 4:**
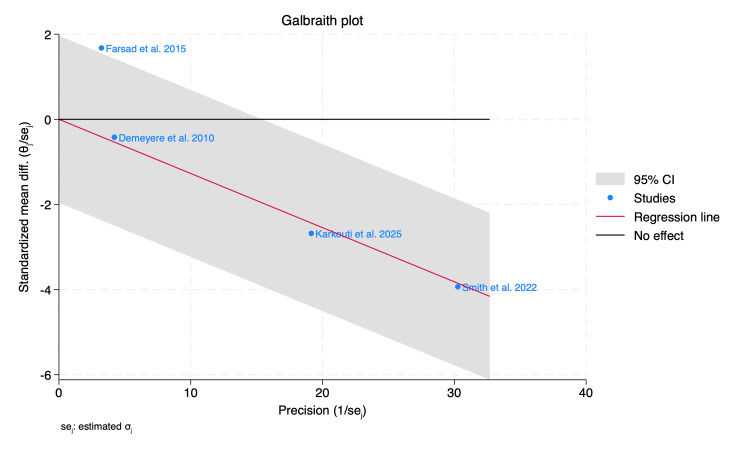
Galbraith plot of mean change of international normalized ratio (INR). Data from [17,18,20,21].

International normalized ratio (INR): Four RCTs assessed the mean change in INR at 24 hours following the procedure. The pooled estimate showed a significant reduction in patients who received PCC compared to FFP by 0.13 (MD = -0.13, 95% CI: -0.18 to -0.07, p < 0.001; I² = 0.00%, p = 0.22), as shown in Figure 2. Furthermore, we performed a Doi plot to investigate possible publication bias, which showed major asymmetry in the distribution of studies, with an LFK index of 4.91, as shown in Figure 3, indicating potential publication bias. On the other hand, all studies were visualized within the 95% precision area of the pooled effect, except for one study, highlighting its documented heterogeneity compared to the others, as shown in Figure 4. 

Secondary Outcomes

**Figure 5:**
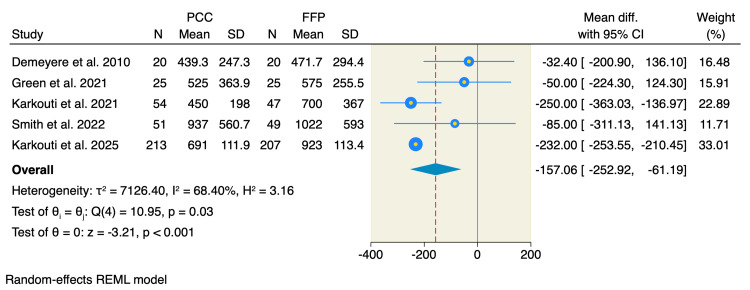
Random-effects model of chest tube drainage volume. Data from [9,17,19-21].

Chest tube drainage: Five RCTs assessed the mean volume of blood loss drained through the chest tube during the procedure. The pooled MD showed a significant reduction in drained blood loss following the administration of PCC compared to FFP, with significant heterogeneity among the reported studies (MD = -157.06 mL, 95% CI: -252.92 to -61.19, p < 0.001; I² = 68.4%, p = 0.03), as shown in Figure 5. Furthermore, we performed a leave-one-out sensitivity analysis, and no single study had a disproportionate effect on the overall pooled estimate (Appendices).

**Figure 6:**
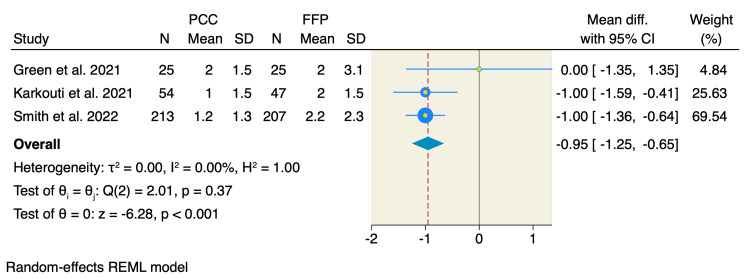
Random-effects model of mean RBC transfusion volume. Data from [9,19,20].

Volume of red blood cell (RBC) transfusion and number of patients: Three RCTs assessed the mean RBC transfusion volume. The pooled MD showed a significant reduction in the required mean RBC transfusion units following PCC compared to FFP (MD = -0.95, 95% CI: -1.25 to -0.65, p < 0.001; I² = 0.00%, p = 0.37) (Figure 6). Moreover, four RCTs reported the number of patients requiring RBC transfusion, of which the rate was 52.9% (179 of 338 patients) in the PCC group compared to 66.5% (215 of 323 patients) in the FFP group. The pooled estimate showed no significant difference between the two groups (RR = 0.87, 95% CI: 0.74 to 1.01, p = 0.07; I² = 0.00%, p = 0.91) (Appendices).

Volume of allogeneic transfusions and hemostatic effectiveness rates: Three RCTs assessed the volume of total allogeneic blood transfusion. The pooled MD showed no significant difference between the two study groups (MD = -4.37, 95% CI: -9.7 to 0.96, p = 0.11; I² = 93.98%, p < 0.01) (Appendices). Meanwhile, the hemostatic effectiveness rate was significantly higher among patients who received PCC, with 76.6% (263 of 343 patients) compared to 57.9% (190 of 328 patients) in the FFP group. The pooled RR showed significantly higher effectiveness rates following PCC administration compared to FFP (RR = 1.18, 95% CI: 1.02 to 1.46, p = 0.03; I² = 0.00%, p = 0.61) (Appendices).

Hospital and intensive care unit (ICU) length of stay (LOS): Four RCTs assessed the mean hospital and ICU LOS of the patients. The pooled estimate showed no significant difference in hospital LOS between patients who received PCC and those in the FFP group (MD = -1.01, 95% CI: -2.61 to 0.59, p = 0.22; I² = 79.14%, p = 0.02) (Appendices). Additionally, the pooled estimate showed no significant difference between the two study groups regarding ICU LOS (MD = -0.01, 95% CI: -0.67 to 0.66, p = 0.98; I² = 29.05%, p = 0.12) (Appendices).

Safety Outcomes

Four RCTs assessed 30-day mortality rates. The event rate was 3.21% (11 of 343 patients) in the PCC group and 3.96% (13 of 328 patients) in the FFP group. The pooled RR showed no significant difference between the two study groups (RR = 0.82, 95% CI: 0.37 to 1.81, p = 0.62; I² = 0.00%, p = 0.98) (Appendices).

Four RCTs assessed reoperation rates for bleeding. A total of 4.53% (14 of 309 patients) in the PCC group and 6.98% (21 of 301 patients) in the FFP group required reoperation to control bleeding. The pooled RR showed no significant difference between the two groups (RR = 0.68, 95% CI: 0.36 to 1.29, p = 0.24; I² = 0.00%, p = 0.97) (Appendices).

Four RCTs assessed thromboembolic event rates, with 7.58% (26 of 343 patients) in the PCC group and 7.31% (24 of 328 patients) in the FFP group. The pooled RR showed no significant difference between the two groups (RR = 1.03, 95% CI: 0.60 to 1.76, p = 0.92; I² = 0.00%, p = 0.76) (Appendices). Additionally, the stroke/TIA event rate was 2.62% (9 of 343 patients) in the PCC group and 3.66% (12 of 328 patients) in the FFP group. The pooled RR showed no significant difference between the two groups (RR = 0.80, 95% CI: 0.33 to 1.92, p = 0.62; I² = 0.00%, p = 0.60) (Appendices).

Three RCTs assessed AKI rates, with 14.78% (47 of 318 patients) in the PCC group and 19.8% (60 of 303 patients) in the FFP group. The pooled RR showed no significant difference between the two study groups (RR = 0.82, 95% CI: 0.50 to 1.36, p = 0.45; I² = 40.9%, p = 0.23) (Appendices).

Discussion

Our meta-analysis of six RCTs including 761 patients assessed the effectiveness of PCC compared to FFP in restoring the hemostatic function of patients undergoing major cardiac surgeries. The present analysis found the following: (1) administration of PCC was associated with significant reductions in INR values compared to FFP; (2) patients who received PCC had less blood drained from chest tubes, shorter hospital stays, and fewer RBC units transfused following the procedure compared to those who received FFP; (3) the hemostatic effectiveness rate was higher in the PCC group compared to FFP; and (4) PCC showed a good safety profile regarding the incidence rates of mortality, reoperation for bleeding, thrombotic events, stroke/TIA, and AKI compared to FFP.

In recent years, the rationale for using PCC has increased in clinical practice, especially for managing warfarin-induced bleeding [22,23]. Moreover, prior clinical evidence has documented the use of PCC in patients undergoing major cardiac surgeries to reduce the incidence of bleeding, RBC transfusions, and bleeding-associated complications [24-26]. Additionally, recent data suggest that PCC might offer clinical benefits by reducing the volume of postoperative RBC transfusions and improving hemostatic function compared to FFP in major cardiac surgeries [27,28]. It is well documented that PCC is biologically more prohemostatic than FFP, as PCC contains procoagulant factors at concentrations up to 25 times higher than those in FFP, with a low concentration of anticoagulants sufficient to prevent hemostatic activation, despite not containing the full array of coagulation factors present in FFP [29].

Our study found that the use of PCC was associated with superior INR correction compared to FFP. While this has been documented in patients receiving warfarin and those with warfarin-related complications, there has been no clear evidence suggesting a superior effect of PCC over FFP in the setting of major cardiac surgeries [30]. Our study confirmed the superior effectiveness of PCC over FFP in this setting, and these findings were consistent with those reported by a major RCT conducted by Smith and colleagues on patients undergoing cardiac surgery with CPB [20], which showed an effect estimate (MD = -0.12, p < 0.001) following PCC administration compared to FFP. The superior effect of PCC over FFP in rapidly correcting INR could be attributed to the higher factor concentrations in PCC, which are known to influence INR values [31]. In addition, the same group conducted a study to assess the magnitude of difference in INR correction and found that greater INR correction was associated with intra- and postoperative reductions in RBC transfusions and shorter ICU stays [32].

An important finding of our study is that the hemostatic effectiveness rate was higher in the PCC group compared to FFP, indicating that more patients in the PCC group did not require additional doses of PCC or allogeneic transfusions compared to those who received FFP. This could be attributed to the fact that PCC is associated with significant reductions in hemodilution necessitating RBC transfusions commonly observed with FFP [20]. This finding is particularly relevant today, as blood banks are often undersupplied. Our finding aligns with previous major RCTs that reported a higher percentage of patients allocated to PCC who avoided any allogeneic blood transfusions on days 1 and 5 postoperatively, compared to none in the FFP group [20,21].

The widespread use of PCC in major cardiac surgeries has raised concerns regarding thrombotic complications. However, current formulations of inactive PCC include a variety of anticoagulants such as heparin, proteins S and C, and antithrombin III, which have the potential to balance or reduce thrombotic risks compared to other formulations [33]. Moreover, the key safety consideration with enhanced hemostatic activity is the incidence of thromboembolic events, making safety assessment a main focus of our meta-analysis. We found no significant difference in thrombotic complication rates, and our findings are supported by prior evidence from recent retrospective trials [25,27] and major RCTs [9,19], all of which reported no increased risk of thromboembolic adverse events. On the other hand, previous retrospective studies have suggested an increased risk of AKI following PCC administration [27,28]. However, we found no significant difference between PCC and FFP regarding AKI rates, consistent with evidence from major RCTs [9,19-21]. Notably, the event rates of AKI were lower than those documented in retrospective studies, indicating the need for further prospective research to confirm the current findings. Additionally, the PCC doses used across the included studies were lower than those reported in retrospective studies and lower than the commonly used doses for warfarin reversal, except for the FARES-II study, which used a dose of 25 IU/kg [21].

Clinical implications 

Our study of six RCTs including 761 patients revealed clinically relevant findings. First, the use of PCC was superior in rapidly correcting INR, with a significant impact on hemostatic effectiveness compared to FFP; thus, the use of PCC in major cardiac surgeries could significantly reduce the provisional use of FFP. Second, the use of PCC in clinical settings could translate into annual savings of FFP units that can be redirected to address the supply challenges associated with plasma-derived products [34]. Furthermore, the higher hemostatic efficacy with a safety profile comparable to FFP will benefit the healthcare system by reducing the rates of major bleeding, allogeneic transfusions, particularly RBCs and platelets, and the use of rescue hemostatic therapies such as recombinant activated factor VII, all of which have been linked to increased risks of major adverse events and overuse of healthcare resources [35-37].

Limitations 

Despite the importance of the current findings, there are limitations that need to be addressed before implementing them into clinical practice. First, there was no standardized transfusion protocol across the included studies; however, all studies adhered to protocols based on current guidelines. Second, baseline variables such as timing of therapy, preoperative hemoglobin and platelet levels, and dosing of heparin or protamine were not documented across all included studies; thus, bleeding management could not be standardized or assessed for variability across studies. Furthermore, dropout rates differed markedly across studies, ranging from 7% to more than 25%, which could limit the robustness of the ITT analysis. Additionally, as there is no reliable or robust measure of hemostatic effectiveness in major cardiac surgeries, the included studies varied in their primary outcomes: some reported hemostatic effectiveness rates as the primary outcome, while others reported change in INR at 24 hours as the primary endpoint. This inconsistency in outcome reporting may raise concerns regarding the power of each analysis. Finally, most included studies excluded patients with recent thrombotic events, which could raise concerns when applying the current findings to clinical practice.

## Conclusions

This meta-analysis of six RCTs demonstrated that PCC significantly improves INR correction and hemostatic effectiveness compared to FFP in major cardiac surgeries, with reduced blood loss and transfusion requirements. Importantly, PCC exhibited a comparable safety profile regarding mortality, thromboembolic events, stroke, and AKI. These findings support the clinical adoption of PCC as an effective, safe, and resource-efficient alternative to FFP in perioperative bleeding management.

## Figures and Tables

**Figure 7:**
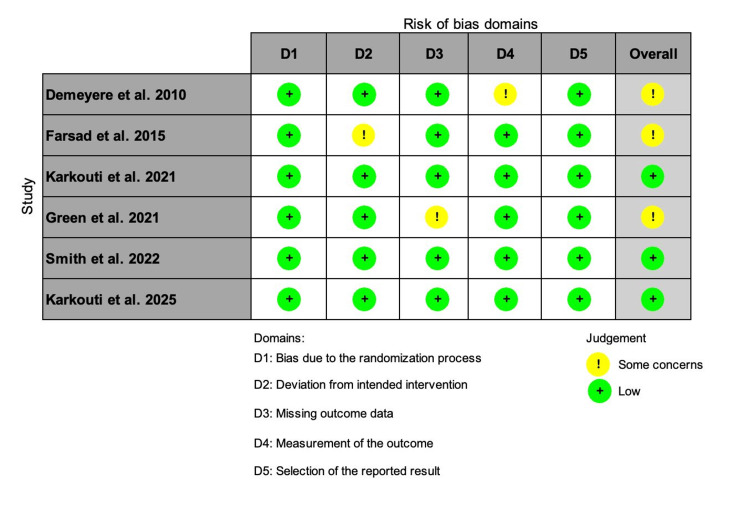
Risk of bias (ROB) assessment of the included studies. Data from [9,17-21].

**Figure 8:**
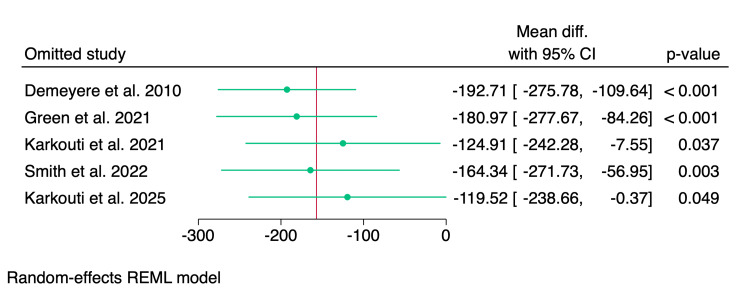
Leave-one-out sensitivity analysis of chest tube drainage. Data from [17-21].

**Figure 9:**
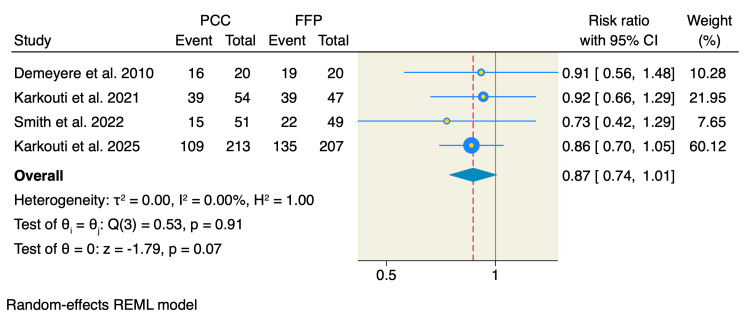
Random-effects model of patients required RBC transfusion. Data from [17,18,20,21].

**Figure 10:**
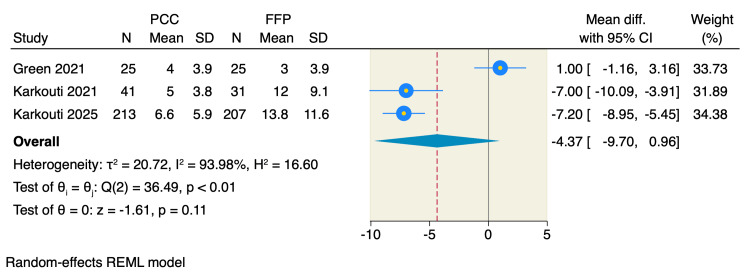
Random-effects model of the total volume of allogenic transfusion. Data from [9,19,21].

**Figure 11:**
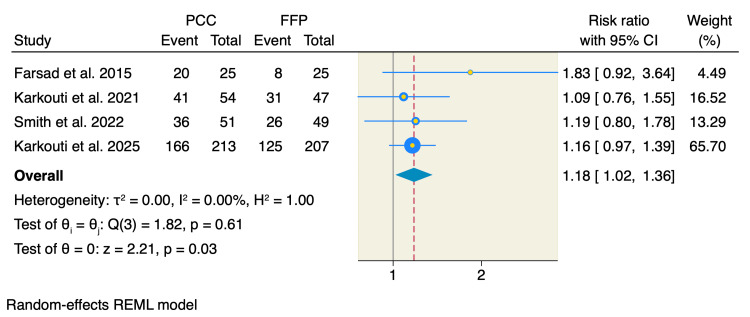
Random-effects model of the hemostatic effectiveness rates. Data from [9,18,20,21].

**Figure 12:**
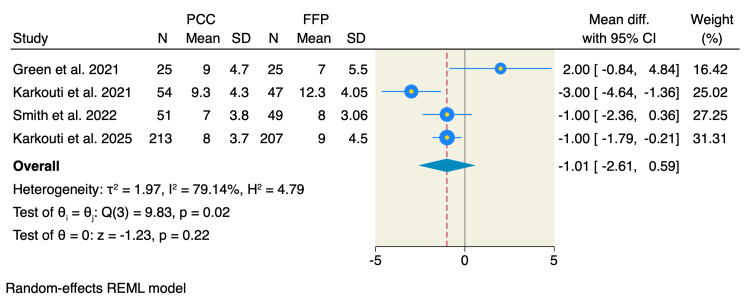
Random-effects model of the hospital length of stay (LOS). Data from [9,19-21].

**Figure 13:**
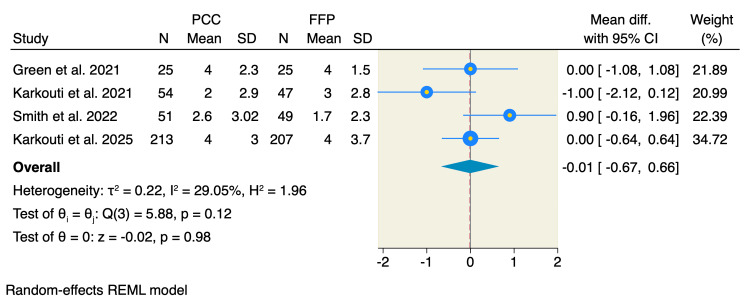
Random-effects model of the ICU length of stay (LOS). Data from [9,19-21].

**Figure 14:**
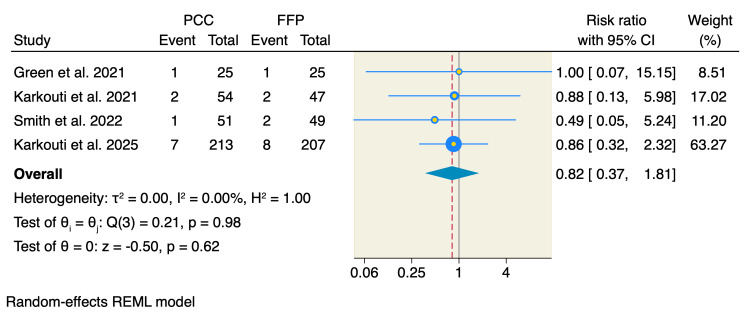
Random-effects model of 30-days mortality rates. Data from [9,19-21].

**Figure 15:**
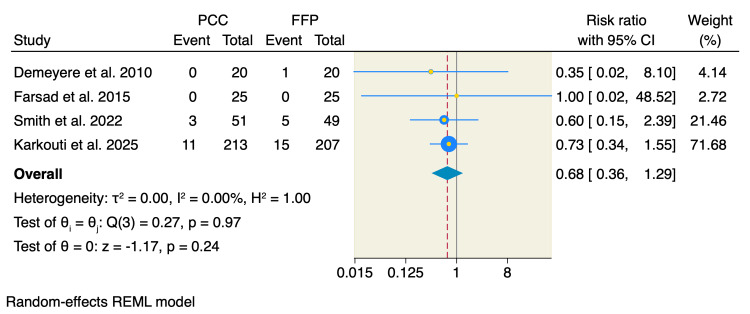
Random-effects model of the rates of reoperation for bleeding. Data from [17,18,20,21].

**Figure 16:**
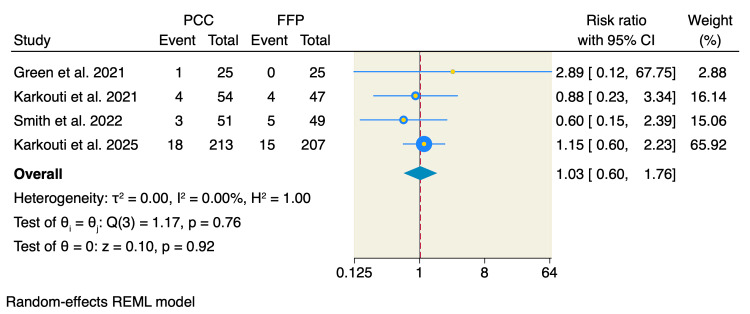
Random-effects model of the thromboembolic events rates. Data from [9,19,20,21].

**Figure 17:**
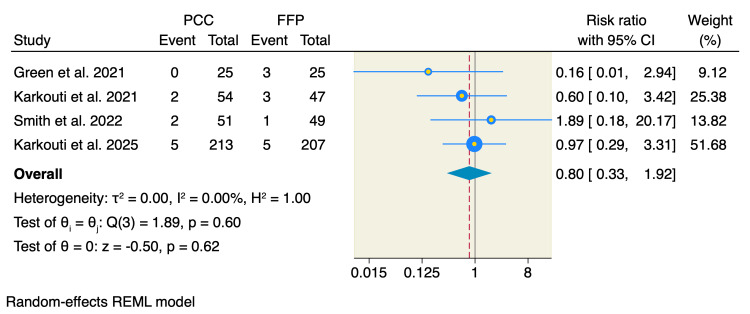
Random-effects model of the stroke/transient ischemic attack (TIA) rates. Data from [9,19,20,21].

**Figure 18:**
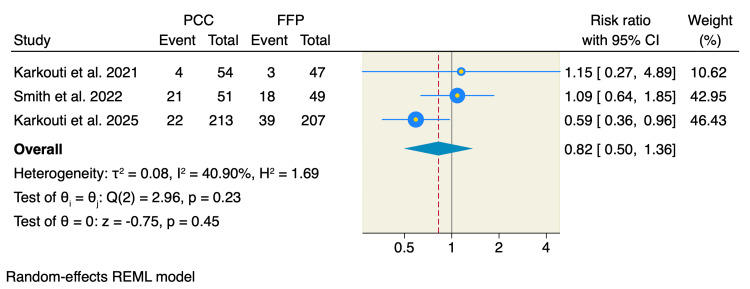
Random-effects model of the rates of acute kidney injury (AKI). Data from [9,20,21].

## Appendices

**Table 3:** Search strategy for each database.

Database	Search Strategy	Records Identified
PubMed	((Prothrombin Complex Concentrate OR Coagulation Factor IX OR Factor IX OR Plasma Thromboplastin Component OR Autoprothrombin) AND (Fresh Frozen Plasma OR Blood Plasma) AND (Cardiac Surgical Procedures OR cardiac surgery))	N = 129
MEDLINE	((Prothrombin Complex Concentrate OR Coagulation Factor IX OR Factor IX Complex OR Plasma Thromboplastin Component OR Autoprothrombin II).mp. AND (Fresh Frozen Plasma OR Blood Plasma).mp. AND (Cardiac Surgery OR Heart Surgery).mp.)	N = 63
Scopus	TITLE-ABS-KEY(("Prothrombin Complex Concentrate" OR "Coagulation Factor IX" OR "Factor IX Complex" OR "Plasma Thromboplastin Component" OR "Autoprothrombin II") AND ("Fresh Frozen Plasma" OR "Blood Plasma") AND ("Cardiac Surgery" OR "Cardiac Surgical Procedures"))	N = 137
Web of Science	TS=(("Prothrombin Complex Concentrate" OR "Coagulation Factor IX" OR "Factor IX Complex" OR "Plasma Thromboplastin Component" OR "Autoprothrombin II") AND ("Fresh Frozen Plasma" OR "Blood Plasma") AND ("Cardiac Surgery" OR "Cardiac Surgical Procedures"))	N = 138
Cochrane CENTRAL	(("Prothrombin Complex Concentrate" OR "Coagulation Factor IX" OR "Factor IX Complex" OR "Plasma Thromboplastin Component" OR "Autoprothrombin II") AND ("Fresh Frozen Plasma" OR "Blood Plasma") AND ("Cardiac Surgery" OR "Cardiac Surgical Procedures")) in Trials	N = 10
